# Robot-assisted arm training in patients with Parkinson’s disease: a pilot study

**DOI:** 10.1186/1743-0003-11-28

**Published:** 2014-03-05

**Authors:** Alessandro Picelli, Stefano Tamburin, Michele Passuello, Andreas Waldner, Nicola Smania

**Affiliations:** 1 Department of Neurological and Movement Sciences, Neuromotor and Cognitive Rehabilitation Research Center, University of Verona, P.le L.A. Scuro, 10, 37134 Verona, Italy; 2 Department of Neurological and Movement Sciences, Neurology Section, University of Verona, Verona, Italy; 3 Degree Course in Physiotherapy, University of Verona, Verona, Italy; 4 "Villa Melitta" Rehabilitation Clinic, Bolzano, Italy; 5 Neurological Rehabilitation Unit, Azienda Ospedaliera-Universitaria Integrata, Verona, Italy

**Keywords:** Rehabilitation, Physical therapy, Basal ganglia

## Abstract

**Background:**

Despite the growing diffusion of robotic devices in neurorehabilitation, no previous study investigated the effects of robotic training on arm impairment due to Parkinson’s disease. The aim of this pilot study was to evaluate whether robot-assisted arm training might improve upper limb function in patients with Parkinson’s disease.

**Findings:**

Ten patients with Parkinson’s disease (Hoehn and Yahr stage 2.5-3) received ten, 45-minute, treatment sessions, five days a week, for two consecutive weeks. Robot-assisted arm training was performed with the Bi-Manu-Track (Reha-Stim, Berlin, Germany) that provides a computer-controlled, repetitive, bilateral, mirror-like practice of forearm pronation/supination and wrist extension/flexion. Patients were trained according to the following modalities: passive-passive (both arms moved by the machine) and active-active (both arms actively moving against resistance). The dominant upper limb was evaluated before and immediately after treatment as well as at two weeks of follow-up. Outcomes were the nine-hole peg test, the Fugl-Meyer assessment (upper limb section) and the Unified Parkinson’s Disease Rating Scale. After treatment, a significant improvement was found in the nine-hole peg test (*P* = 0.007) as well as in the upper limb section of the Fugl-Meyer assessment (*P* = 0.012). Findings were confirmed at the 2-week follow-up evaluation only for the nine-hole peg test (*P* = 0.007). No significant improvement was found in the Unified Parkinson’s Disease Rating Scale at both post-treatment and follow-up evaluations.

**Conclusions:**

Our findings support the hypothesis that robot-assisted arm training might be a promising tool in order to improve upper limb function in patients with Parkinson’s disease.

## Introduction

Parkinson’s disease (PD) is an idiopathic neurodegenerative disorder due to a progressive loss of dopaminergic neurons in the substantia nigra pars compacta [1]. Typical features of PD are bradykinesia (slowed movement), hypokinesia (poverty of movement), rigidity and resting tremor [1,2]. Even if impaired manual dexterity with progressive limitations in reaching, grasping and fine motor tasks has been described in PD, to date evidence base for upper limb intervention strategies in parkinsonian patients is lacking [3].

Robotic arm training (RAT) was found to effectively improve upper limb function in patients with neurological disorders, such as stroke [4]. As to PD, forced use, task-specific, intensive, training programs based on robotic devices were found to effectively improve lower limb function [5-10]. Despite the growing diffusion of robotic devices in neurorehabilitation, to date no previous study investigated the effects of robotic training on arm impairment due to PD. The aim of this pilot study was to evaluate whether RAT might improve upper limb function in patients with PD.

## Methods

This study was performed in the Neurorehabilitation Unit of the Azienda Ospedaliera-Universitaria Integrata of Verona, Italy. Inclusion criteria: confirmed diagnosis of idiopathic PD according to the UK Brain Bank Criteria [11]; Hoehn and Yahr (H&Y) stage of 2.5 or 3 determined in the "on" phase [12]; Mini Mental State Examination score >24 [13]. Exclusion criteria: severe dyskinesias or "on-off" fluctuations; change of PD medication during the study; deficits of somatic sensation involving the upper limbs; other neurological or orthopedic conditions involving the upper limbs.

All participants were outpatients and gave their informed written consent for participation in the study, which was carried out according to the Declaration of Helsinki and was approved by the Ethics Committee of the Department of Neurological and Movement Sciences of Verona University.

During the study, participants were instructed to take their normal PD medications: they were tested and trained during the on phase (1 to 2.5 hours after taking morning dose). Participants did not perform any type of rehabilitation in the three months before the study, nor undergo any form of training other than that scheduled in the study protocol.

### Treatment procedures

Each patient underwent a training program consisting of ten, 45-minute sessions (including rest periods), five days a week (from Monday to Friday) for two consecutive weeks.

**Figure 1:**
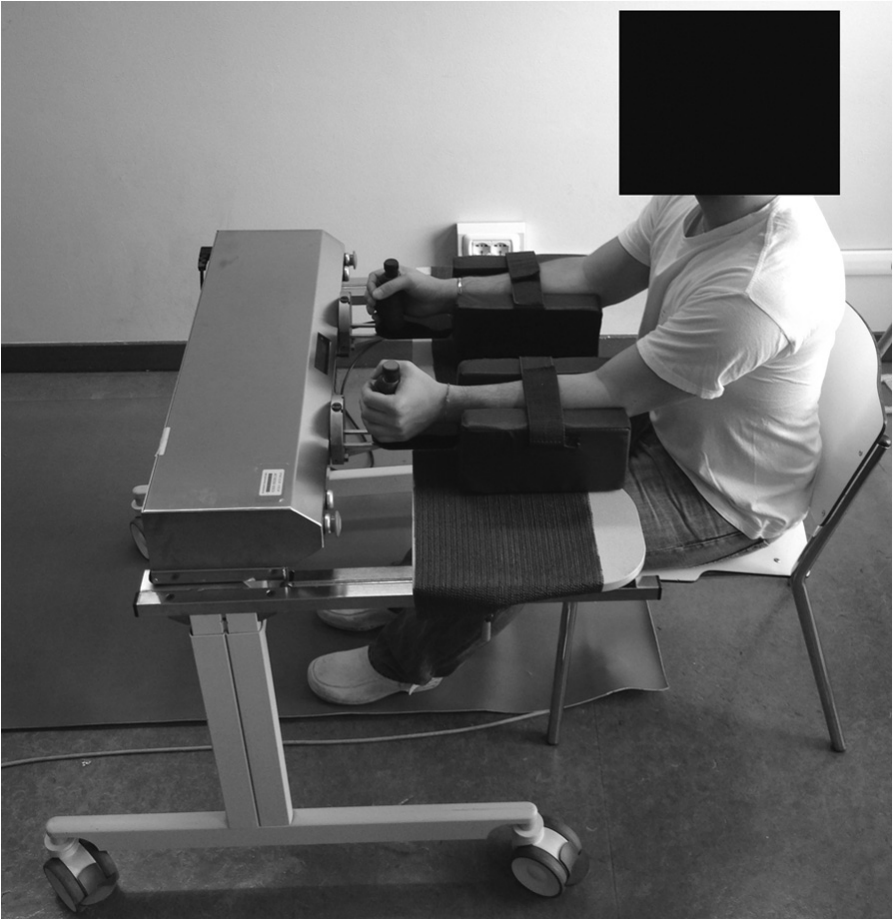
Robot-assisted arm training.

Robot-assisted arm training was performed with the Bi-Manu-Track (Reha-Stim, Berlin, Germany) that provides a computer-controlled, repetitive, bilateral, mirror-like practice of forearm pronation/supination and wrist extension/flexion according to three modalities: passive-passive (both arms moved by the machine), active-passive (one arm driving the other), and active-active (both arms actively moving against resistance) [14]. As shown in Figure 1, patients sat at a height-adjustable table with their elbow bent at 90°, putting their forearms into an arm trough and grasping a handle (written informed consent for the publication of this image was obtained). Each training session consisted of two parts with a 5-minute rest between them. First we trained forearm pronation/supination for 20 minutes: 10 minutes of passive-passive mode (100 repetitions) followed by 10 minutes of active-active mode (100 repetitions). Then we trained wrist extension/flexion for 20 minutes: 10 minutes of passive-passive mode (100 repetitions) followed by 10 minutes of active-active mode (100 repetitions). Amplitude and resistance were set individually.

### Testing procedures

Patients were evaluated before (T0), immediately after treatment (T1) (primary endpoint) and at two weeks of follow-up (T2). The same rater evaluated all patients.

#### Outcome measures

The nine-hole peg test (NHPT) was used to assess dominant hand dexterity. We required patients to take 9 pegs from a container placing them into 9 holes on a board and vice versa as quickly as possible. Score was the time taken to complete the test activity [15].

The Fugl-Meyer assessment (FM) was used to evaluate dominant upper limb motor ability to perform selective movements. The FM upper limb section allows a maximum score of 66, with sub-scores of 36 for the upper arm, 10 for the wrist, 14 for the hand, and 6 for coordination and speed of movement [16,17].

The Unified Parkinson’s Disease Rating Scale (UPDRS) was used to measure disease severity in PD. It has three subscales: I–mentation, behavior, and mood (range 0–16); II–activities of daily living (range 0–52); III–motor examination (range 0–108). Total score is the sum of these subscales (range 0–176) [18,19].

### Statistical analysis

Statistical analysis was carried out with SPSS 21.0 (SPSS Inc, Chicago, IL). The Friedman test was used to analyze overall changes in performance between the different evaluation sessions. In the presence of significant main effects, the Wilcoxon signed ranks test was used on the T1 vs. T0, T2 vs. T0 and T2 vs. T1 comparisons to determine any significant difference. Descriptive analysis was used to evaluate the effect size measures (Cohen’s *d* calculation) and the 95% confidence intervals [20]. The level for significance was *P* < 0.05. The Bonferroni correction was used when investigating multiple comparisons (*P* < 0.016) [21].

## Results

Ten right-handed subjects (7 males and 3 females; mean age 70.7 years) presenting with idiopathic PD (mean duration 7.1 years) were recruited from among 18 outpatients consecutively admitted to our Neurorehabilitation Unit from April to October 2012. No dropout was observed. No adverse event occurred during the study. Row data (medians and interquartile ranges) of patients’ performance at T0, T1 and T2 evaluations are reported in Table 1.

**Table 1:** Row data of patients’ performance in all outcome measures

**Outcomes**	**Before treatment**	**After treatment**	**Follow-up**
	**Median (IQR)**	**Median (IQR)**	**Median (IQR)**
**Nine-hole peg test** (s)	16.20 (15.80 to 18.18)	13.80 (13.06 to 14.92)	13.80 (13.08 to 15.13)
**Fugl-Meyer assessment** (0–66)	60.00 (57.25 to 62.00)	65.00 (62.50 to 65.75)	63.50 (62.00 to 65.75)
**UPDRS motor examination** (0–108)	16.00 (12.25 to 18.50)	14.50 (13.25 to 15.75)	15.00 (13.25 to 16.00)
**UPDRS total** (0–176)	34.50 (25.27 to 37.75)	30.00 (24.00 to 34.00)	27.50 (23.00 to 34.00)

*Abbreviations*: *IQR* Interquartile range, *s* seconds, *UPDRS* Unified Parkinson’s Disease Rating Scale.

As to the NHPT, a significant overall change was found between the three time points (*P* = 0.007; χ^2^ = 9.800). A significant difference was found at both T1 vs. T0 (*P* = 0.007) and T2 vs. T0 (*P* = 0.007) comparisons. As to the FM, a significant overall change was found between the three time points (*P* = 0.003; χ^2^ = 11.400). A significant difference was found only at T1 vs. T0 (*P* = 0.012) comparison. As to the UPDRS, no significant overall change was found between the three time points (*P* = 0.062; χ^2^ = 5.568) at the Friedman test. Treatment effects in all outcome measures are reported in Table 2.

**Table 2:** Treatment effects in all outcome measures

**Outcomes**	**Comparisons**			**95% Confidence interval**		
	**Wilcoxon signed ranks test**			**(Effect size)**		
	**T1 vs. T0**	**T2 vs. T0**	**T2 vs. T1**	**T1 vs. T0**	**T2 vs. T0**	**T2 vs. T1**
	***P *** **value (Z)**	***P *** **value (Z)**	***P *** **value (Z)**	**(*r*)**	**(*r*)**	**(*r*)**
**Nine-hole peg test** (s)	0.007 (-2.701)*	0.007 (-2.701)*	0.359 (-0.918)	1.90 to 4.78 (-0.60)	1.73 to 4.34 (-0.53)	-1.07 to 0.47 (0.08)
**Fugl-Meyer assessment** (0–66)	0.012 (-2.527)*	0.018 (-2.371)	0.606 (-0.516)	-6.31 to -1.28 (0.45)	-5.53 to -1.26 (0.41)	-1.62 to 2.42 (-0.07)
**UPDRS motor examination** (0–108)	0.097 (-1.658)	0.174 (-1.358)	0.334 (-0.966)	-0.34 to 2.54 (-0.14)	-0.40 to 2.00 (-0.11)	-1.05 to 0.45 (0.04)
**UPDRS total** (0–176)	0.046 (-1.995)	0.037 (-2.082)	0.813 (-0.214)	0.26 to 6.73 (-0.20)	0.54 to 6.65 (-0.20)	-1.30 to 1.50 (-0.01)

*Abbreviations:UPDRS* Unified Parkinson’s Disease Rating Scale.

* = statistically significant after Bonferroni correction (*P* < 0.016).

## Discussion

Our results show that ten, 45-minute sessions of RAT may improve fine and gross motor function of the dominant upper limb in patients with PD (H&Y 2.5-3). As measured by the NHPT, improvements of fine motor function were maintained also at the follow-up examination. Conversely, no significant change was found in the UPDRS.

In people with PD, altered upper limb function generally manifests as impaired timing and force modulation, progressively affecting the quality of hand movement [3]. Even if intensive, task-specific, practice has been proposed to reduce arm impairment due to PD, the most effective rehabilitative approach in order to best facilitate upper limb skill learning has not yet been defined [3]. Our preliminary findings about the role of RAT in PD are in keeping with those evidences about the effective use of robotic devices provide task-specific, intensive, training programs in patients with progressive lower limb functional impairment due to PD [5-10]. Furthermore, our findings are in line with those of Lee and colleagues, which examined the effects of constraint-induced movement therapy in twenty patients with PD (H&Y 2–3), observing significant improvements of fine and gross motor performance of the upper limb after twenty, 3-hour, treatment sessions [22].

In order to understand why RAT showed to improve upper limb function in PD, we hypothesize that several repetitions of rhythmic arm movements could act as an external proprioceptive cue, by reinforcing the neuronal circuits that contribute to the upper limb movements. In particular, RAT provides an external rhythm that could improve motor output bypassing the deficient internal motor generation system (including the supplementary motor area and basal ganglia) that would support the generation on actions based on intention and internal reference frame [22]. In addition, it is plausible that the strengthening effect of RAT would play a role. In line with this issue, a previous case series study by Combs and colleagues, described a significantly reduction of disability as well as an improvement of quality of life after a training program based on 24 to 36 boxing sessions in 6 patients with PD (H&Y 1–4) [23]. Unfortunately, the Authors did not evaluate upper limbs function before and after treatment [23]. Thus, we cannot directly compare their data with ours.

This pilot study was limited by the lack of a control group and the small sample size. Furthermore there is no long-term follow-up evaluation and no assessment of activities of daily living.

## Conclusions

Our preliminary findings support the hypothesis that RAT might be a promising tool in order to improve upper limb function in patients with PD. However, there is the possibility that changes observed in this pilot study might be due to a placebo effect. Furthermore, it would be useful to evaluate RAT not only in terms of effectiveness but also in terms of costs and time taken to prepare the treatment setting. On these bases, future, properly sized, randomized controlled trials dealing with RAT compared to conventional/non-robotic rehabilitation are needed in order to further validate our results.

## Abbreviations

PD: Parkinson’s disease; RAT: Robotic arm training; H&Y: Hoehn and Yahr; NHPT: Nine-hole peg test; FM: Fugl-Meyer assessment; UPDRS: Unified Parkinson’s Disease Rating Scale.

## Competing interests

The authors declare that they have no competing interest.

## Authors’ contributions

AP conceived the study, made substantial contribution to its design, performed the statistical analysis and drafted the manuscript. ST participated in the design of the study and helped to draft the manuscript. MP carried out the acquisition of data. AW revised critically the manuscript for important intellectual content. NS coordinated the study and revised critically the manuscript for important intellectual content. All authors read and approved the final manuscript.

## References

[B1] Meara J Koller WC Parkinson’s disease and Parkinsonism in the elderly 2000 Cambridge: Cambridge University Press

[B2] National Institute of Health and Clinical Excellence Parkinson’s disease: diagnosis and management in primary and secondary care 2006 London (UK): Royal College of Physicians 21089238

[B3] Quinn L Busse M Dal Bello-Haas V Management of upper extremity dysfunction in people with Parkinson disease and Huntington disease: facilitating outcomes across the disease lifespan J Hand Therin press 10.1016/j.jht.2012.11.001 23231827

[B4] Mehrholz J Hädrich A Platz T Kugler J Pohl M Electromechanical and robot-assisted arm training for improving generic activities of daily living, arm function, and arm muscle strength after stroke Cochrane Database Syst Rev 2012 6 CD006876 10.1002/14651858.CD006876.pub3 22696362

[B5] Lo AC Chang VC Gianfrancesco MA Friedman JH Patterson TS Benedicto DF Reduction of freezing of gait in Parkinson’s disease by repetitive robot-assisted treadmill training: a pilot study J Neuroeng Rehabil 2010 7 51 10.1186/1743-0003-7-51 20946640 PMC2972300

[B6] Picelli A Melotti C Origano F Waldner A Fiaschi A Santilli V Smania N Robot-assisted gait training in patients with Parkinson disease: a randomized controlled trial Neurorehabil Neural Repair 2012 26 353 361 10.1177/1545968311424417 22258155

[B7] Picelli A Melotti C Origano F Waldner A Gimigliano R Smania N Does robotic gait training improve balance in Parkinson’s disease? A randomized controlled trial Parkinsonism Relat Disord 2012 18 990 993 10.1016/j.parkreldis.2012.05.010 22673035

[B8] Carda S Invernizzi M Baricich A Comi C Croquelois A Cisari C Robotic gait training is not superior to conventional treadmill training in parkinson disease: a single-blind randomized controlled trial Neurorehabil Neural Repair 2012 26 1027 1034 10.1177/1545968312446753 22623206

[B9] Picelli A Melotti C Origano F Neri R Waldner A Smania N Robot-assisted gait training versus equal intensity treadmill training in patients with mild to moderate Parkinson’s disease: a randomized controlled trial Parkinsonism Relat Disord 2013 19 605 610 10.1016/j.parkreldis.2013.02.010 23490463

[B10] Smania N Picelli A Geroin C Munari D Waldner A Gandolfi M Robot-assisted gait training in patients with Parkinson’s disease Neurodegen Dis Manage 2013 3 321 330 10.2217/nmt.13.34

[B11] Hughes AJ Daniel SE Kilford L Lees AJ Accuracy of clinical diagnosis of idiopathic Parkinson’s disease: a clinicopathological study of 100 cases J Neurol Neurosurg Psychiatry 1992 55 181 184 10.1136/jnnp.55.3.181 1564476 PMC1014720

[B12] Hoehn MM Yahr MD Parkinsonism: onset, progression and mortality Neurology 1967 17 427 442 10.1212/WNL.17.5.427 6067254

[B13] Folstein MF Folstein SE McHugh PR Mini-mental state: a practical method for grading the cognitive state of patients for the clinician J Psychiatr Res 1975 12 189 198 10.1016/0022-3956(75)90026-6 1202204

[B14] Hesse S Schulte-Tigges G Konrad M Bardeleben A Werner C Robot-assisted arm trainer for the passive and active practice of bilateral forearm and wrist movements in hemiparetic subjects Arch Phys Med Rehabil 2003 84 915 920 10.1016/S0003-9993(02)04954-7 12808550

[B15] Oxford Grice K Vogel KA Le V Mitchell A Muniz S Vollmer MA Adult norms for a commercially available nine hole peg test for finger dexterity Am J Occup Ther 2003 57 570 573 10.5014/ajot.57.5.570 14527120

[B16] Fugl-Meyer AR Jääskö L Leyman I Olsson S Steglind S The post-stroke hemiplegic patient. 1. a method for evaluation of physical performance Scand J Rehabil Med 1975 7 13 31 1135616

[B17] Lee KS Lee WH Hwang S Modified constraint-induced movement therapy improves fine and gross motor performance of the upper limb in Parkinson disease Am J Phys Med Rehabil 2011 90 380 386 10.1097/PHM.0b013e31820b15cd 21389845

[B18] Fahn S Elton RL Fahn S, Marsden CD, Goldstein M, Calne DB UPDRS program members. Unified Parkinsons Disease Rating Scale Recent developments in Parkinsons disease, Volume 2 1987 Macmillan Healthcare Information: Florham Park, NJ 153 163

[B19] Shulman LM Gruber-Baldini AL Anderson KE Fishman PS Reich SG Weiner WJ The clinically important difference on the unified Parkinson’s disease rating scale Arch Neurol 2010 67 64 70 20065131 10.1001/archneurol.2009.295

[B20] Cohen J Statistical power analysis for the behavioral sciences 1988 2 Lawrence Erlbaum: Hillsdale, NJ

[B21] Bryant TN Machin D Wilson BA, McLellan DL Statistical methods Rehabilitation studies handbook 1997 Cambridge, UK: Cambridge University Press 189 204

[B22] Nieuwboer A Rochester L Müncks L Swinnen SP Motor learning in Parkinson’s disease: limitations and potential for rehabilitation Parkinsonism Relat Disord 2009 15 Suppl 3 53 58 20083008 10.1016/S1353-8020(09)70781-3

[B23] Combs SA Diehl MD Staples WH Conn L Davis K Lewis N Schaneman K Boxing training for patients with Parkinson disease: a case series Phys Ther 2011 91 132 142 10.2522/ptj.20100142 21088118

